# Ninth International Medical Congress, Washington, D. C., September, 1887

**Published:** 1887-11

**Authors:** 


### NINTH INTERNATIONAL MEDICAL CONGRESS, WASHINGTON, D. C., SEPTEMBER, 1887.

### SECTION XVIII, DENTAL AND ORAL SURGERY.

Reported for the Independent Practitioner, by "Mrs. M. W. J."

Continued From Page 536.

### TUESDAY MORNING SESSION.

Secretary Dudley read a translation of the paper of Dr. E. Brasseur, of Paris, France, entitled "The use of Air in Dental Therapeutics."

The paper was an exposition of the value to the dentist of hot air and remedial agents, applied by means of the Thermo-injector.

He said that the sensitiveness of dentine depends on hygrometric conditions. Nerve filaments penetrate the dentine, some points being so sensitive that it is impossible to touch them without causing extreme suffering. These points he terms "true ganglionic centres." The teeth are the guardians of the alimentary canal, and capable of appreciating the nature of substances submitted to them for mastication, distinguishing promptly between gravel, chalk, sugar, acids, fatty matters, etc., being thus perfect organs of feeling, hyperæsthesia being the pathological state. The use of arsenical paste to allay this sensitiveness, he condemns as extremely dangerous. Teeth with scarcely any decay are often found with fistulous abscesses, with no apparent cause for the death of the pulp until the patient explains that the tooth was very painful when cut, and the dentist put in something which cured it, and this "something" we recognize as arsenious paste. Crystals of carbolic acid, used for the same purpose, by imbibition, destroy the living matter of the tooth substance. If, however, carbolic acid is heated in a tube to 42° C., by means of the Thermo-injector, the heated vapor will penetrate the canaliculi and destroy micro-organisms, producing an anæsthetic instead of a caustic action. If the dentine is extremely sensitive, rendering cutting intolerable, a mixture of eugenol 10 gr., muriate of cocaine, 1 gr., veratria, 10 centigrammes, dissolved in alcohol, adding tannin 25 and glycerine 8 grains, will be found very soothing when applied to the tooth with blasts of heated air. In the several stages, where dentine is discolored and partially softened and the pulp nearly exposed, the work of destruction, unless promptly checked, will continue until the pulp dies and alveolar abscess follows with all its train of complications. To avert this, we use the heated air, exhausting all moisture; apply bi-chloride of mercury 1 to 1000, followed by oil of cloves or eugenol, with blasts of hot air; cover the walls with copal varnish and paraffine, equal parts, drying with hot air, and place in the bottom of the cavity an asbestos wafer soaked in oil of cloves, and fill with oxy-phosphate.

When a pulp is first exposed, either by caries or traumatism, it is but slightly sensitive. As disease progresses it becomes first sensitive to cold only; then to heat and cold both, and in the last stage heat alone is painful. Thus we have the key to the condition of the pulp. The first stage requires most delicate treatment in the endeavor to preserve the delicate pulp. The patient wants immediate relief; if pain continues he shrinks from every touch, and probably demands that the pulp be killed. Every effort should be used to make him understand the value of that which he is anxious to destroy, and the importance of preserving the vitality of the tooth; that a tooth, if dead, is held in place only by the ligaments of the pericemental membrane; it has no force to struggle against subsequent attacks of disease, while nature, abhorring dead matter, will make every effort to get rid of it. Therefore, every endeavor should be used to preserve it alive. If caries has penetrated to the pulp chamber, it must be kept free from moisture and septic agents, certain to follow the presence of saliva in the cavity. Put on the rubber-dam with clamps; dry out with spunk, followed by hot air. Capping the pulp is perfectly feasible, but is a very delicate operation, requiring great dexterity; all compression is fatal. Blasts of heated air drive the pulp back if hernia has occurred through afflux of blood, moisture causing it to swell. Under the influence of hot air we can see it draw back like an earth worm. In many cases a disk of platinum can be placed over the pulp, but this is not always practicable. Rosenthal's pulpine, or oil of cloves and anhydrous oxide of zinc made into a paste of the consistency of cream, may be applied directly, and the cavity filled with oxy-phosphate of zinc, with an amalgam surface.

In the third stage the pulp has reached the point which necessitates devitalization. If gangrene has already occurred, we recognize it promptly by the odor, the root canals being filled with putrescent matter. We know but little of the ferment in dead pulps, or the microbes of caries. We leave that to the microscopists. With the aid of heated air we avoid interminable dressings, and the complications which may arise. Applying the rubber-dam, dry with absolute alcohol and apply bi-chloride of mercury, or biniodide, which is more powerful. Anæsthetic vapors of chloroform, ether, or sulphide of carbon, under pressure of hot air, are very penetrating, and attract the water which is normally present, a small amount of vapor eliminating a comparatively large quantity of water ; these vapors do not tarnish the brilliancy of the enamel, so precious in the front teeth. The use of these vapors, and those of carbolic acid and iodoform, transfer a large number of cases called incurable to the ranks of conservatism, and materially contract the limits of extraction. As long as any putrefactive elements remain, caries will go on and pus-formation continue. One or two drops of carbolie acid in the root canal, followed by soft gutta-percha, will drive all pus in the canal through the apex to the fistulous opening, where bubbles will emerge, showing that it has penetrated. Clearing the root canal and opening the apex with a root trimmer, tannin and iodoform can be placed in the canula of the injector and blown through the apex by compressed hot air, to the walls of the abscess sac. Then placing a wad of felt at the apex, stop with gutta-percha. The cure is complete when the wad is odorless, when the permanent filling may be inserted. In chronic periodontitis, a douche of hot air on the neck of the tooth penetrates to the bottom of the affected part, and affords prompt relief. Hot air gives gutta-percha great plasticity, renders amalgam more plastic, hastens the setting of oxy-phosphate fillings, and is invaluable in drying the surface of gold fillings if accidentally wet by saliva or the breath. Paraffine should be melted, or copal varnish painted on the surface of cement fillings, to prevent disintegration, often enabling teeth which were mere wrecks to do good service.

Correct diagnosis is of the greatest importance, as doubt endangers the life of the pulp; alternate blasts of hot or cold air afford a sure test of the condition of the pulp. The Thermo-injector is invaluable for the introduction of bleaching agents, causing them to penetrate every portion of the discolored dentine ; also for the application of remedial vapors in diseases of the antrum and in catarrh.

The hour for adjournment having arrived, the discussion of this paper was postponed.

TUESDAY AFTERNOON SESSION.

The discussion of Dr. Brasseur's paper was opened by a written paper from Dr. C. A. Brackett, of Newport, Rhode Island. He said that, having had the opportunity of studying the paper in the original, he was perhaps better prepared to judge of its merits than those who had listened to a translation of portions of a paper which, as a whole, was too long for the time assigned to it. The prominent idea of the paper was that of lessening sensitiveness of dentine by hot air, in the process of drying eliminating the normally-contained water of the tissues, thus diminishing, however, its capacity for either normal or pathological functions, one which is that of conveying sensation. Moisture can, without injury, be eliminated from the walls of a cavity which does not approach the pulp, or from the dentine of a pulpless tooth, the next step being the introduction of antiseptic agents. The author of the paper recognized the different pathological conditions of the pulp; his views of pulp-capping are not new to us. Hygrometric filling material is not always objectionable; it is sometimes both comfortable and successful, absorbing the slight exudation from the pulp surface. Great credit is due the author for the apparatus invented, though the advantage of blowing in dry medicinal powders over liquids, as conveyed by the syringe, is doubtful. "Dead teeth" are spoken of by the translator where pulpless teeth, with the pericemental connection intact, were intended by the author.

(The paper was illustrated by drawings of the apparatus, so constructed as to utilize heat generated either by gas or petroleum, or by electricity.)

Dr. Truman, of Philadelphia, said that in the use of hot air, as of any other agent, it must be borne in mind that the tooth is not a dead body. The pulp having prolongations through the dentine, heat, if carried past a certain degree, might destroy those delicate ramifications, or convey such irritation to the pulp as to cause its death.

Dr. W. H. Morgan, of Nashville, thought the statements in the paper too broad, and the language not sufficiently exact. He did not see the advantage of first thoroughly drying to desiccation, and then filling with liquid antiseptics, neither did he admit that absolute dryness was essential to the success of a gold filling. Submarine fillings that were put in thirty or forty years ago, before the day of rubber-dam, did good service. He also took exception to the broad ground taken by the author as to the treatment of alveolar abscess. If the cause was removed and putrefaction arrested, nature would, in a large majority of cases, effect a cure without any treatment at our hands.

Dr. Geo. Whitefield, of Evansville, Ind., uses a rapidly revolving, keen bur in the excavation of sensitive dentine. Discolored dentine he bleaches by the liberation of chlorine from common salt by the action of electricity. Brine penetrates the tubuli, and chlorine, being liberated, whitens the dentine very rapidly.

Dr. S. H. Guilford, of Philadelphia, considered the use of hot air a valuable adjunct, and described in detail the construction of an apparatus for the storage of compressed air, and the mode of conveying it by tubes to the side of the chair, where it can be used either hot or cold as desired, the effects of hot air being very satisfactory to both patient and operator, especially in excavating sensitive dentine and in preparing roots for the reception of dowel-teeth.

Dr. A. H. Brockway, of Brooklyn, thought such elaborate apparatus frightfully cumbersome and absolutely needless, the chip syringe and warm air from the alcohol lamp accomplishing the same results. He had abandoned the desiccating system because of danger to the pulp. He now uses for sensitive dentine very sharp burs, kept moist by a stream of tepid water.

(TO BE CONTINUED.)

